# Evolution in Dentistry

**Published:** 1890-04

**Authors:** J. A. Robinson


### Evolution in Dentistry.

BY J. A. ROBINSON, D.D.S.

Man perceives everything in this material world with the sensuous faculties first, next by his rational faculties, and thence upward, to seeing by spiritual discernment, and these faculties correspond to the mineral, vegetable and animal life below our own in this material world. There are also degrees of altitude—our higher faculties—just as strong, that teach us the moral law, the loving our neighbors as ourselves, and like the trees that have wood and blossom and fruit; three degrees of life, each distinct from the other but combined to make a grand harmonious whole. Now we reach this highest life through internal processes, as all life comes down from the great source of *all life* by evolution and reaction. We only perceive it when reaction takes place, which is the desire to perfect things, or make them better. When the process takes place in the material world, or in deeds of charity, any act to systematize labor, to improve machinery, to shorten hours or relieve suffering, it is evolution—and the object of evolution is *use*. Mr. Conway claims that Mr. Emerson was first to suggest evolution, and quotes the famous couplet from Emerson's volume of nature,

> " And striving to be man, the worm
> Mounts through all the spires of form."

The first theory of decay of teeth was as wide from fact as the ancient idea of the sun rising in the east and sailing around the world to go down in the western horizon; it was an apparent truth. Harwood and Tucker taught that decay first began within the dentine and came to the outside from influences within; Prof. Watt demonstrated the acid theory forty years ago, and Dr. Miller, of Berlin, has shown us the germs and cultivated them, so we are certain the germ theory is correct. The acid theory and the germ theory are both true, and demonstrate the law of all life, viz: that all life comes from reaction. It took forty years to arrive at the truth; as long as the children of Israel were going to the promised land. We have also been shown that fermentation and putrefaction are the two most prolific sources of decay. If that be true then, cleanliness must be a potent factor in preventing decay. To preserve the teeth when decay has once commenced, the cavities must be hermetically sealed, and people must be taught the value of cleanliness. This was no part of the theory or practice sixty years ago. Some people had learned that sulphuric acid would remove the black that gathered upon the teeth, and I have seen whole sets of teeth utterly destroyed by its too frequent use.

In the summer of 1850 I went into some financial speculations, in which I lost almost all the accumulations of fifteen years' hard work. After a struggle of two years in trying to redeem my shattered fortunes I gave it up and determined to take up with Horace Greeley's advice and go west; so in 1852 I came to Cleveland, Ohio, where my brother B. F. Robinson, was engaged in dentistry with Dr. Ambler. I had become a first-class toothmaker and had manufactured block ivory for quite a number of dentists in Boston, and was admitted a partner in Cleveland on equal terms, only I was to manufacture all the teeth used in the concern. In the fall of 1852—25th of November—I landed in Cleveland and began work. I was full of faith that I could make again the money I had lost, nearly or quite fifteen thousand dollars. I knew I had a big load to carry, for we had eight children, and I brought my aunt, who had lived with us twenty years, to assist in taking care of the young family, also a nephew who was learning dentistry and who was good help in the laboratory. So we had more of a family than old John Rogers had when he was burned at the stake. Cleveland then contained about 45,000 people. My advent in Cleveland was hailed with delight by the liberal dentists, and we soon formed a city dental society. It was here that I first met Dr. Atkinson—God bless his good heart!! for he proved himself a true friend in time of need and great distress. In July or August, 1853, I came down with fever and ague as did also nearly every one of my family. I struggled against it until I gave out and was sick three months of billious fever. I was so reduced that I weighed only one hundred and eighteen pounds when I could walk out of the house while my nominal weight was one hundred and seventy-five. Atkinson never failed to come and visit me every morning and bring me some little delicacy on his way to his office. The good angel that was always in his heart when there was distress anywhere directed his steps to my door and the words of courage and generous deeds of kindness will never be forgotten by myself or any of my family. We had formed a city society, as I said before, and I had given illustrations of my method of filling with pellets and the graver-pointed plugger, and Atkinson remarked after I had been showing the new plan that he should say when I was dead that "Jere Robinson, before the Cleveland Dental Society, gave the first clinics in filling teeth that were ever given in the world."

Atkinson was just bringing out the mallet. Old Dr. Merritt, with whom Atkinson had worked, was from Pennsylvania, and filled teeth with a mallet. I did not take to the mallet at first; I was strong and thought hand pressure was best. Crystal gold was in its infancy and could not be made as serviceable as crystal gold foil to-day. So I worked at times with Atkinson, malleting for him. It was hard to give up hand pressure, I confess; I was a little egotistical, for as water seeks its level, so man, driven by circumstances out of the plane of his desires will return to his native instincts at the first opportunity. I kept filling with pellets a long time after the mallet was introduced. My graver-pointed plugger is the *one* of the serrated point, as the lever is the *one* of all mechanical powers; the wedge is only a double lever and the screw an inclined plane of the lever and only called by that name for convenience to designate what kind of a lever is needed for *use*, as all mathematics is a multiplication of the unit or its division.

As you, my friend Prof. Taft, requested me to give a sort of autobiography of myself in these pages, I will digress and insert a letter from my son Jere E. Robinson, who had access to some facts regarding my ancestry that I did not possess. I insert the whole letter.

CLEVELAND, O., Feb. 7th, 1890.

DEAR FATHER:—I find upon examination of the Cogswell biography the following:

John Cogswell and family came to America in 1635, sailing from Bristol, Engand, on May 23d, in ship Angel Gabriel, and were wrecked at Pemiquad, in Maine, August 15th, of same year. The ship in which they made their eventful voyage and lost most of their earthly possessions, had a history about as eventful as that of the passengers on her final voyage. She was built for Sir Walter Raleigh by Sir Charles Snell, and upon the attainder of Sir Walter was forfeited, costing Sir Charles almost his entire estate and livings.

Sir Walter Raleigh also made his second and last voyage, just previous to his execution, in 1618, in the same ship. She had a glorious name but a checkered career.

In looking up your connection with the Cogswell family, I find much of interest before they came to America, and it would tickle your pride to know that I have traced it back to Lord Humphrey Cogswell (1447), and could give you the coat of arms of the family. I will just mention that such is a fact, and then pass to the American history of the family, as you know I am an American clear through, and being one further remove from the Cogswell than you, have not so much interest in their illustrious ancestry.

John Cogswell, the ancestor of the American family, was born in 1592, in Westbury Leigh, County of Wilts, England. At the age of 23 he married the daughter of the parish vicar, succeeded to her father's business, and settled down in the old homestead. Shortly after his marriage his parents died and he inherited the "Mylls at Repind," the homestead, etc. He was a large manufacturer of broadcloths and kerseymeres, and gave the mills a reputation which they have retained to the present day.

The factories occupy the same location and are owned and managed by Cogswells, and as recently as the last World's Fair, at Vienna, took first premiums. Why he sold out and came to this (at that time) wilderness was for much the same reason that prompted you to give up an established business and pleasant home in New England and settle in the large and growing west, —viz : better opportunities for a large and growing family, and belief that a new country offered better chances for distinction than an old-settled and nobility-ridden country could do, even when the party leaving was reasonably prosperous at home. Though he lost all of his worldly possessions, and had to start anew, history shows that he was right, and that he was the direct ancestor of two of the presidents of the United States, through the female line, and also that in a direct line Ralph Waldo Emerson, Oliver Wendel Holmes, Elisha Whittlesey, M.C., and within the memory of myself, at the head of the Treasury Department of the United States, and the Supt. of the building of the Washington Monument until relieved of that duty by death in 1865, at the ripe age of 82 years; John Wentworth, L.L.D., Wm. S Robinson (" Warrington ") the famous correspondent of the *Springfield Republican* during the war of the Rebellion and the turbulent times immediately succeeding, Eldridge Gerry Robinson, one of the pioneer abolitionists of this country and many others of equal note but whose names are not accessible at this time.

You are connected with the Cogswells on both father and mother's side, but more directly by your mother, who was Martha Cogswell, daughter of Emerson Cogswell, who descended from John as follows: William 2d, William 3d, Emerson 4th, Emerson 5th—your grandfather. On your father's side your grandmother was Susanna Cogswell, who was a daughter of Emerson 4th, William 3d, William 2d, John, and I also find your father was connected with the Cogswells by blood in another remove about the third generation. In fact, the Robinsons of your family and Cogswells descended from John, are about as nearly related as brother and sister, and could never have married under our present laws. So far as I have been able to judge no harm has resulted, either physically or mentally from the union, but 'tis a risky experiment and in my judgment quite reprehensible. But things were quite different in those days. The Cogswells moved after a sojourn of one or two generations in Ipswich, Mass., to Concord, known as "Old Concord," where there were but few settlers, and if the boys and girls would get married they must take, as Cain did, those of near blood. Would you like to know how the above-named celebrities descended from the same stem as yourself? To commence with the one who has attained the highest honor a republic can bestow, Prest. William Henry Harrison married a daughter of Reverend Timothy Symmes, who was Lydia Cogswell, a daughter of Francis 4th, John 3d, William 2d, John 1st. You have to reach back to William 2d to find kinship with the present president of the United States, from whom you differ so radically in a political way, and as I think I hear you say I have enough of the Cogswell blood in me to feel no pride in the kinship.

Rufus Choate was not by blood a Cogswell as you have supposed. His father's first wife was Mary Cogswell, who lived but a few months after marriage and bore him no children. W. Choate's mother was a Miriam Foster, daughter of Capt. Aaron Foster, whose name I am unable to find, but as through three generations the Cogswells and Fosters married and lived in Chebacco Parish, Ipswich, Mass., through all these years, it is fair to presume that Capt. Amos Foster was in part a Cogswell.

With Ralph Waldo Emerson, *the grandest of them all*, the lineage is clear and direct, and like yourself, comes down on both father and mother's side until he loses the name in Emerson, which again turns back to Cogswell in the second generation. Hannah Cogswell, daughter of John 1st, married Deacon Cornelius Waldo, a farmer for John Cogswell, and from this line came the name of Waldo, so prominent in Emerson's name. It is hard to trace R. W. Emerson through the changes back and forth into the Cogswell family, but he without doubt (yourself and brothers excepted) was the best representative of the Cogswells not having the name.

Now, my dear father, I think I have shown enough of lineage to satisfy a not very ambitious man, and will stop. Modesty forbids my saying much about my immediate ancestry, but should I be spared to write after you have followed the long line of ancestry so well represented in the names I have mentioned, I may have something to say concerning one who is the peer of any and dearer to me far than this long list of names I know only of as I read and as the world prizes them.

You have pioneered in dentistry and worked up the literature as well as the science of what has grown to take place among the learned professions, and it can—when all your work is done—be truthfully said, as of yore, "Well done, thou good and faithful servant."

